# The Disturbing Effect of the Stray Magnetic Fields on Magnetoimpedance Sensors

**DOI:** 10.3390/s16101723

**Published:** 2016-10-17

**Authors:** Tao Wang, Yong Zhou, Chong Lei, Shaotao Zhi, Lei Guo, Hengyu Li, Zhizheng Wu, Shaorong Xie, Jun Luo, Huayan Pu

**Affiliations:** 1 School of Mechatronics Engineering and Automation, Shanghai University, Shanghai 200072, China; wangt@shu.edu.cn (T.W.); lihengyu@shu.edu.cn (H.L.); zhizhengwu@shu.edu.cn (Z.W.); srxie@shu.edu.cn (S.X.); luojun@shu.edu.cn (J.L.); 2 Key Laboratory for Thin Film and Microfabrication of Ministry of Education, Department of Micro/Nano Electronics, School of electronic information and electrical engineering, Shanghai Jiao Tong University, Shanghai 200240, China; shangwujingshen@163.com (Y.Z.); leiqhd@sjtu.edu.cn (C.L.); zhist1987@sjtu.edu.cn (S.Z.); g2252330@sjtu.edu.cn (L.G.)

**Keywords:** disturbing effect, detect, giant magnetoimpedance, stray magnetic fields

## Abstract

The disturbing effect of the stray magnetic fields of Fe-based amorphous ribbons on the giant magnetoimpedance (GMI) sensor has been investigated systematically in this paper. Two simple methods were used for examining the disturbing effect of the stray magnetic fields of ribbons on the GMI sensor. In order to study the influence of the stray magnetic fields on the GMI effect, the square-shaped amorphous ribbons were tested in front, at the back, on the left and on the top of a meander-line GMI sensor made up of soft ferromagnetic films, respectively. Experimental results show that the presence of ribbons in front or at the back of GMI sensor shifts the GMI curve to a lower external magnetic field. On the contrary, the presence of ribbons on the left or on the top of the GMI sensor shifts the GMI curve to a higher external magnetic field, which is related to the coupling effect of the external magnetic field and the stray magnetic fields. The influence of the area and angle of ribbons on GMI was also studied in this work. The GMI sensor exhibits high linearity for detection of the stray magnetic fields, which has made it feasible to construct a sensitive magnetometer for detecting the typical stray magnetic fields of general soft ferromagnetic materials.

## 1. Introduction

The giant magnetoimpedance (GMI) effect is the large variation of impedance in soft magnetic materials when subjected to an external magnetic field [1,2,3,4]. Considerable efforts have been made to develop highly sensitive GMI sensors over the past two decades. It has been demonstrated that GMI sensors based on multilayered films are highly sensitive to the external magnetic field [1,2,3,4,5,6,7,8,9], thus making it ideal for use in detecting the stray magnetic fields.

It is a well-known fact that the soft ferromagnetic amorphous ribbons have been widely applied in the field of magnetic cores, transformers, motors, choke coils and magnetic sensors because of their excellent soft magnetic properties. In general, the amorphous ribbons can produce a stray magnetic field after being magnetized by an external magnetic field, which can cause a disturbance on the magnetic field distribution when it is put around a magnetic sensor, and thus can be detected by the magnetic sensor. In mass production, some of the soft ferromagnetic products may have defects such as cracks, fractures and holes, which can induce different stray magnetic fields. Hence, study of the disturbing effect of stray magnetic fields is very useful for GMI-based nondestructive testing. Up to now, there have been very few studies of the disturbing effect of the stray magnetic fields on the GMI. In this work, the disturbing effect of the ribbons’ stray magnetic fields on the GMI was studied under different conditions, including different areas (1 × 5, 2 × 5, 3 × 5, 4 × 5 and 5 × 5 mm^2^), positions (left, top, back, front) and angles (0°, 45°, 90°) of ribbons, which makes it possible to construct a micro-sized magnetometer with a high linearity for detection of the stray magnetic fields.

## 2. Experimental Details

**Figure 1:**
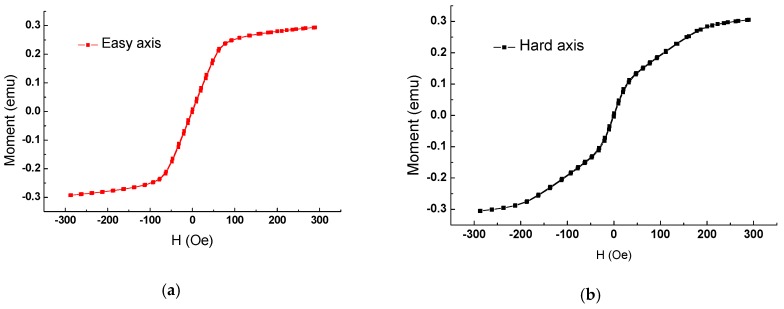
The magnetic hysteresis loop of the NiFe film: (**a**) easy axis (**b**) hard axis [11].

Two GMI-based sensors (sensor I and sensor II) (Fabricated in Key Laboratory for Thin Film and Microfabrication of Ministry of Education) fabricated by Micro-Electro-Mechanical-Systems (MEMS) technology were used in this work, and the detailed fabrication procedure has been described elsewhere [10]. During the production of the sensor, a constant external magnetic field was applied along the transverse direction of the films. Thus, the easy direction is the transverse direction, and the hard direction is the longitudinal direction. Sensor I (NiFe line length: 5 mm, NiFe line width: 0.16 mm, NiFe thickness: 0.003 mm, Cu line length: 4.79 mm, Cu line width: 0.12 mm, Cu thickness: 0.002 mm, line space: 0.06 mm, area of sensitive elements: 5 mm × 4.34 mm) and sensor II (NiFe line length: 5 mm, NiFe line width: 0.16 mm, NiFe thickness: 0.004 mm, Cu line length: 4.79 mm, Cu line width: 0.12 mm, Cu thickness: 0.006 mm, line space: 0.06 mm, area of sensitive elements: 5 mm × 1.26 mm) are both composed of sandwich films (NiFe/Cu/NiFe) with a meander-shape structure. The NiFe film was tested by a Vibrating Sample Magnetometer (Lake Shore Cryotronics, Inc., Westerville, OH, USA), and it is clear that NiFe film (Figure 1) possesses high saturation magnetization, a clear easy axis and a hard axis [11], which are well-suited for use as magnetic sensitive elements. In addition, a 10 µm thick positive photoresist was coated on the sensitive elements for use as a protective layer.

**Figure 2:**
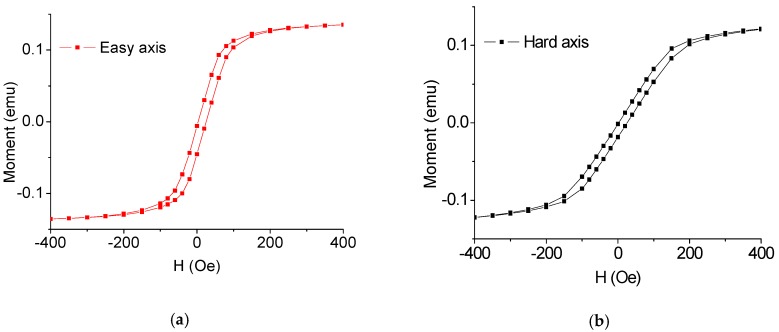
The magnetic hysteresis loop of the amorphous ribbon: (**a**) easy axis (**b**) hard axis.

Fe-based commercial amorphous ribbons (Metglas 2605S, Conway, SC, USA) were purchased from the Metglas Company, possessing a thickness of about 22 µm. In this work, the amorphous ribbons were fabricated into oblong-shaped samples with different areas (1 × 5, 2 × 5, 3 × 5, 4 × 5 and 5 × 5 mm^2^) by MEMS technology. The preparation of the oblong-shaped samples started with a glass wafer on which a single-layer AB glue was spun, and then an Fe-based amorphous ribbon (45 × 60 mm^2^) was pasted on the surface of the glass wafer. After drying it in the air for 24 h, a 10 µm positive photoresist was spun on the ribbon. Then, ultraviolet exposure was performed on the positive photoresist, followed by rinsing it in the developer for 2.5 min. After washing and blow-drying the glass wafer, it was then put into an oven for solidification of the photoresist at 120 °C for 90 min, followed by soaking it in an acidic mixture (HNO_3_, HCl, H_2_O_2_ and H_2_O) with a certain proportion for 5–10 min. Finally, the oblong-shaped samples were obtained after removing the photoresist. From Figure 2, one can find that the ribbon has very good soft magnetic characteristics.

**Figure 3:**
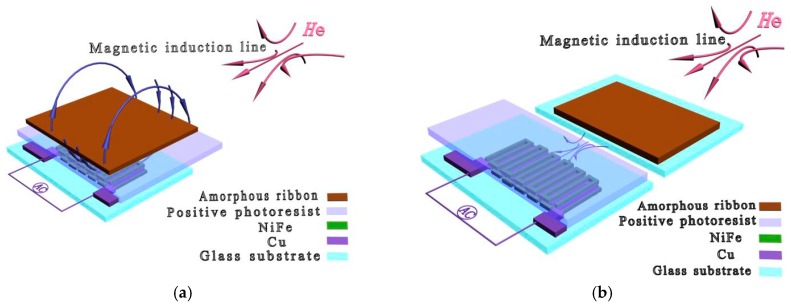
Two simple methods for detection of the stray magnetic fields of soft ferromagnetic amorphous ribbons: (**a**) the ribbon is immobilized on the top of the giant magnetoimpedance sensor and (**b**) the ribbon is immobilized in front of the giant magnetoimpedance sensor. Adapted from [9].

An experimental setup has been used to detect the stray magnetic fields of the soft ferromagnetic amorphous ribbons as shown in Figure 3. Transverse and longitudinal directions were defined as the directions that were perpendicular and parallel to the long axis of the sensitive elements, respectively. GMI sensor I was placed horizontally to connect an impedance analyzer (Agilent E4991A, Agilent, CA, USA) via two Cu electrodes extended from the sensitive elements. During the test, an external magnetic field is applied along the longitudinal direction of the films. The applied magnetic field is perpendicular to the easy axis. An alternating current (AC) of 10 mA over the frequency (*f*) range of 1–50 MHz flows through the films along the longitudinal direction, thereby generating a transverse AC magnetic field determining the changes of the transverse magnetization. When the amorphous ribbons (1 × 5, 2 × 5, 3 × 5, 4 × 5 and 5 × 5 mm^2^) were tested on the top of GMI sensor (Figure 3a), the free sides of the ribbons were facing the sensitive elements, and the distance from the sensitive elements to the top ribbon was about 10 μm (thickness of positive photoresist), respectively. When the amorphous ribbons were tested in front of GMI sensor (Figure 3b), the free sides of the ribbons were placed in parallel with the sensitive elements, and the distance from the sensitive elements to the marginal ribbon was about 2 mm (edge width of the glass substrate). 

A nonuniform external magnetic field (*H*_e_) of 0–80 Oe provided by a solenoid was applied in the longitudinal direction in order to change the transverse permeability in magnetic sensitive elements through modifying the penetration depth of AC. Meanwhile, the ribbon would be magnetized by the longitudinal external magnetic field and thus produce a measurable stray magnetic field, exhibiting the disturbing effect on the magnetic field distribution near the sensitive elements, thereby altering the magnetoimpedance of the sensor and providing a detection signal in the form of a voltage change. The relative change in impedance (GMI ratio) is defined as:

(1) $$\mathrm{GMI}\;\mathrm{ratio}=\Delta Z/Z=100\%\times \frac{Z(H)-Z(H_{max})}{Z(H_{max})}$$

where Z(*H*) and Z(*H*_max_) represent the impedance under the external magnetic field (*H*), and under the maximum external magnetic field (*H*_max_), respectively.

## 3. Results and Discussion

**Figure 4:**
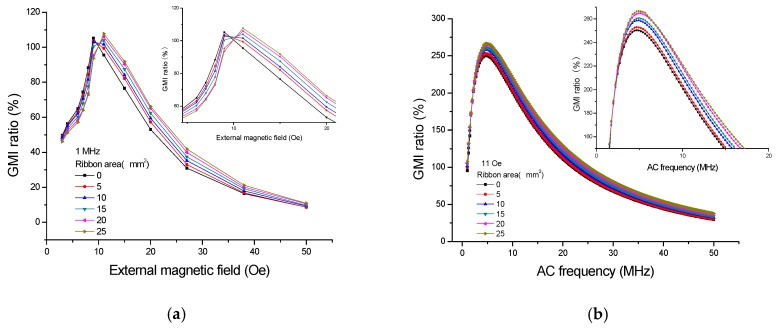
The giant magnetoimpedance responses when the ribbons are tested on the top of the giant magnetoimpedance sensor I: (**a**) field dependence and (**b**) frequency dependence.

Figure 4 shows the GMI responses of the ribbons tested on the top of GMI sensor I; quite evidently, the presence of ribbons has caused the changes in GMI ratio. As can be seen from Figure 4a, the great changes in GMI ratio (*f* = 1 MHz) have taken place at or near the two peak fields (9 Oe and 11 Oe), and this is because the transverse permeability has risen considerably due to the strong rotational magnetization occurred around the anisotropy field. Thus, high detection sensitivity can be obtained around the maximal GMI ratio. [2,4] The largest change (15.54%) in GMI ratio is obtained at *H*_e_ = 8 Oe for 5 × 5 mm^2^ ribbon. The small changes of GMI ratio at low external magnetic fields (<4 Oe) may be attributed to the low-level magnetization in the ribbons. Moreover, the small changes of GMI ratio occur at higher external magnetic fields (>35 Oe), which is related to the stray magnetic fields becoming strongly overwhelmed by the overlarge external magnetic field. [9] It is clear from Figure 4a that the GMI ratio firstly declines (<9 Oe) and then enhances (>9 Oe) due to the presence of the ribbons, namely, the GMI curve shifts to a higher external magnetic field in the presence of ribbons. Furthermore, it is worthwhile to note that the GMI ratio decreases with increasing ribbon area by the application of the external magnetic field of 4–9 Oe but increases with increasing ribbon area by the application of the external magnetic field of 11–35 Oe. Therefore, it is feasible to quantify the stray magnetic fields of the ribbons in the field range of 4–35 Oe. After magnetizing the soft ferromagnetic ribbon on the sensor by the external magnetic field, the ribbon will generate two free magnetic poles and produce a stray magnetic field (Figure 3a) due to its ferromagnetism [12,13], which suggests that the stray magnetic field can partly cancel out the external magnetic field near the sensitive elements. Hence, the GMI curve shifts to a higher external magnetic field in the presence of ribbon. This result differs from that reported by Phan et al. [14] who found an overall increase in GMI ratio after coating a 50 nm thick Co film on the soft ferromagnetic amorphous ribbons. The difference in results is attributed to the different methods. In the early study [14], the Co film was sputtered directly on the amorphous ribbon (contact-type testing), thus resulting in an overall effect. Phan et al. suggest that the presence of the Co layer not only reduces the stray magnetic fields due to surface irregularities, but also closes up the magnetic flux path, both of which contribute to the enhanced GMI effect in Co-coated ribbons. A similar phenomenon was also found in a cobalt ferrite layer-coated ribbon by Phan et al. [15] However, in the current study, the amorphous ribbon is not directly coated on the sensitive elements, and there is 10 µm positive photoresist between the amorphous ribbon and the sensitive elements (non-contact-type), thus resulting in a non-overall effect. Park et al. [16] also observed an enhanced GMI effect in the ion-irradiated Co-based amorphous ribbon, which can be interpreted in terms of the permeability variation associated with domain wall dynamics. The obtained result also differs from results reported in previous works in which the presence of magnetic particles caused an overall enhancement [17,18,19,20] or decline [11,21,22,23] of GMI ratio through the whole external magnetic field.

It also can be seen from Figure 4b that large changes of GMI ratio (*H*_e_ = 11 Oe) can be observed at high frequency. Fundamentally, it is believed that the GMI effect is attributed to combinations of the skin effect and the field dependence of the effective permeability associated with some particular domain structures [2,4], and the impedance is inversely proportional to the skin depth. In the soft magnetic thin film, the skin depth can be expressed as:

(2) $$\delta =\frac{C}{\sqrt{4\pi^{2}f\sigma \mu }}$$

where *C* is the speed of light, *δ* is skin depth, *σ* is the electrical conductivity, *μ* is the effective permeability, and *f* is the frequency of AC. The high-frequency current (>5 MHz) just results in small skin depth, and the large transverse permeability can be obtained due to not only the domain wall motion but also the spin rotation [2,4], both of which contribute to the GMI effect. In other words, the GMI sensor has high field-sensitivity at high frequency and can be highly sensitive to the stray magnetic fields, thereby resulting in large changes of GMI ratio in the presence of the ribbons. The detectable signal grows weaker with increasing frequency (>30 MHz), which might be because the effective permeability becomes smaller due to the single contribution from the spin rotation mechanism.

**Figure 5:**
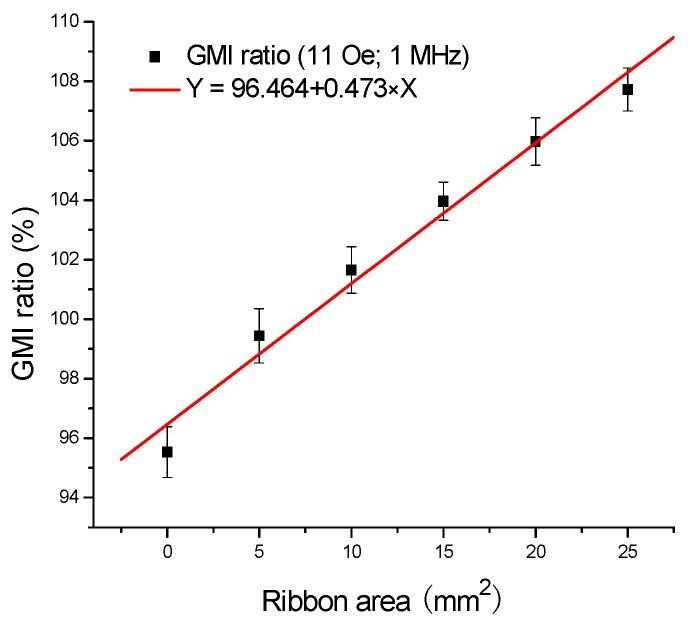
The typical linear relationship between the giant magnetoimpedance ratio and the ribbon area when the ribbons are tested on the top of the giant magnetoimpedance sensor I.

Figure 5 displays the typical linear relationship between the GMI ratio and the ribbon area when the ribbons are tested on the top of the GMI sensor. As can be seen from Figure 5, the GMI ratio increases with the ribbon area from 0 to 25 mm^2^, the regression equation is Y = 96.464 + 0.473 × X, and *R* = 0.990, and the GMI response is linearly proportional to the ribbon area, namely proportional to their stray magnetic field. Consequently, the quantification of the stray magnetic fields of amorphous ribbons was achieved by the GMI sensor.

**Figure 6:**
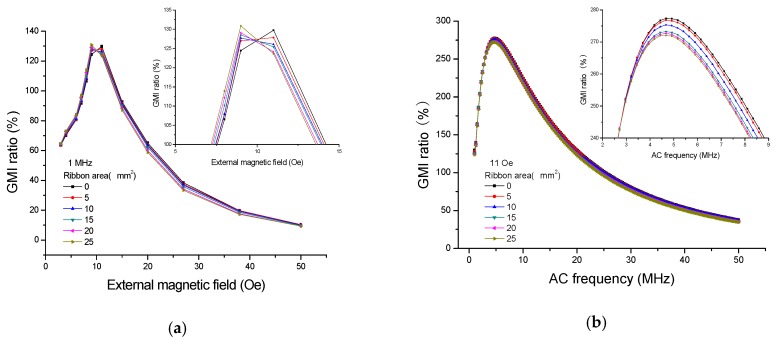
The giant magnetoimpedance responses when the ribbons are tested in front of the giant magnetoimpedance sensor I: (**a**) field dependence and (**b**) frequency dependence.

Figure 6 shows the results of testing the ribbons in front of GMI sensor I. At *f* = 1 MHz, an enhanced GMI effect can be found in Figure 6a when *H*_e_ < 9 Oe. Nevertheless, a reduced GMI effect occurs at *H*_e_ > 9 Oe, and this trend is totally contrary to that reported in Figure 4a. From the magnetic induction distribution of the stray magnetic field in Figure 3b where the ribbon is immobilized in front of GMI sensor in longitudinal direction, it can be qualitatively determined that the longitudinal external magnetic field near the sensitive elements should be strengthened by the stray magnetic field. Therefore, the GMI curve shifts to a lower magnetic field. This result is similar to that reported in our previous study [9] in which the GMI effect firstly enhances and then declines in the presence of soft ferromagnetic NiFe films. The field distribution of the film is similar to that of the ribbon, both of which play a role in strengthening the longitudinal external magnetic field when testing them in front of GMI sensor. Significant changes can be also observed near the maximum GMI ratio in Figure 6a. However, the impedance changes in Figure 6a are much smaller than that in Figure 4a. This is because the distance (about 10 µm) between the sensitive elements and the top ribbon is much smaller than the distance (about 2 mm) between the sensitive elements and the marginal ribbon. Therefore, the top stray magnetic fields are much stronger than the marginal stray magnetic fields, thereby leading to larger changes of GMI ratio in the presence of top ribbons. Moreover, the distance between the sensitive elements and the marginal ribbon can be decreased to a lower extent by cutting the glass substrate down, and a much smaller detectable area or higher GMI response may be achieved. 

**Figure 7:**
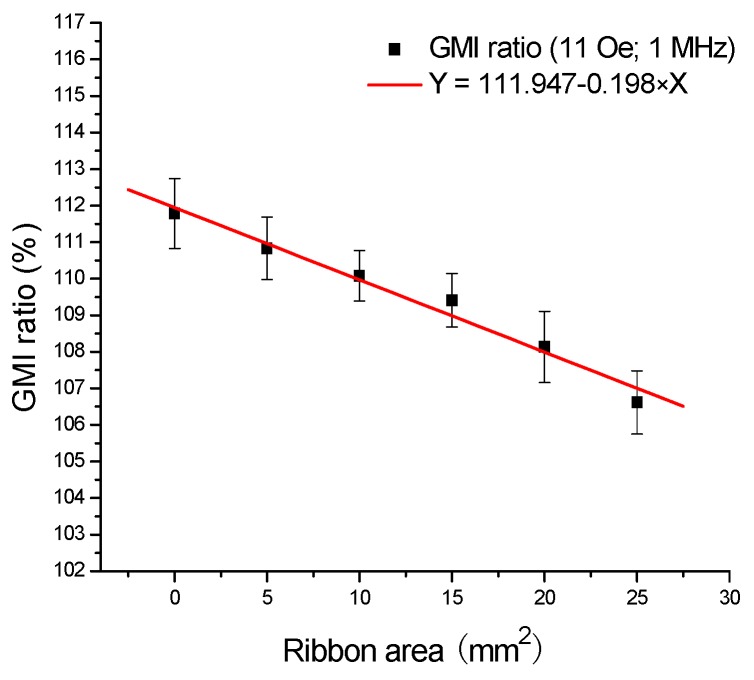
The calibration plots of the giant magnetoimpedance ratio versus the ribbon area when the ribbons are tested in front of the giant magnetoimpedance sensor I.

Figure 6b displays the frequency dependence of GMI ratio (*H*_e_ = 11 Oe) when the ribbons are tested in front of the GMI sensor, obviously, high detection sensitivity can be obtained at high frequency (>5 MHz). As you can see Figure 4b, when *f* < 5 MHz, the six points stay close to each other, and the GMI signals caused by the ribbons are very weak. When 5 MHz < *f* < 30 MHz, clear changes of GMI ratio can be observed in this frequency range, the large change of GMI ratio almost reaches 20% at *f* = 10 MHz when the ribbon (5 × 5 mm^2^) is tested on the top of GMI sensor. When *f* > 30 MHz, the GMI signals caused by the ribbons begin to drop off, which is probably related to the domain wall motion inhibited by the eddy current effect [2,4]. On the other hand, the change of current density for the small area of flowing current and all the related effects are also probably the causes of the reduced GMI effect. Figure 7 shows the calibration plots of GMI ratio versus the ribbon area when the ribbons are tested in front of GMI sensor. The corresponding regression equation is Y = 111.947 − 0.198 × X, and *R* = −0.988. As can be seen in Figure 7, there is a linear calibration between the GMI ratio and the ribbon area from 0 to 25 mm^2^. Moreover, the reproducibility tests were performed on all the samples for five successive measurements, and the relative standard deviation is smaller than 2.0%, indicating a good repeatability. In short, the GMI sensor possesses high linearity and good stability for detection of the stray magnetic fields of Fe-based amorphous ribbons. 

**Figure 8:**
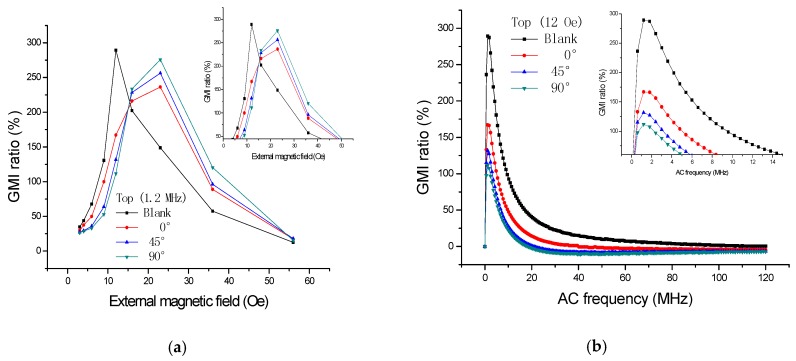
The giant magnetoimpedance responses when the ribbon (3 × 5 mm^2^) is detected on the top of the giant magnetoimpedance sensor II with different angles (0°, 45°, 90°): (**a**) field dependence and (**b**) frequency dependence.

The supplemental tests were performed by using GMI sensor II and Impedance Analyzer (E4990A, Agilent, CA, USA). We have measured the GMI response (Figure 8) when the ribbon (3 × 5 mm^2^) was rotated for different angles (0°, 45°, 90°) on the top of GMI sensor II. There is a 10 µm positive photoresist between the sensitive element and the ribbon during the test. As can be seen from Figure 8a, the GMI curves (0°, 45°, 90°) all move to a higher magnetic field due to the presence of the ribbon on the top of GMI sensor. The larger the degree, the higher field the curve moves to. It can also be explained by the magnetic superimposed effect that the ribbon is actually always magnetized in the longitudinal direction. Even if it is rotated at different angles (0°, 45°, 90°), all the top stray magnetic fields partly cancel out the external magnetic field, and, therefore, all the GMI curves move to a higher field. The larger the degree, the stronger the stray magnetic field, and the more area the ribbon covers on sensitive elements. Furthermore, the longitudinal anisotropy field also exhibits a positive impact on the stray magnetic field.

**Figure 9:**
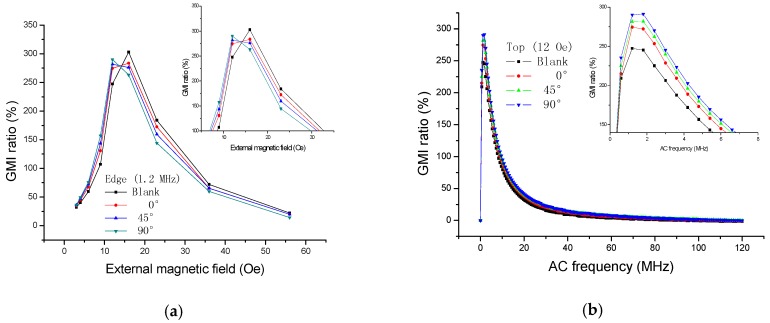
The giant magnetoimpedance responses when the ribbon (3 × 5 mm^2^) is detected in front of the sensor II with different angles (0°, 45°, 90°): (**a**) field dependence and (**b**) frequency dependence.

We have also measured the GMI response when the ribbon (3 × 5 mm^2^) was placed by 0°, 45°, and 90° in front of GMI sensor II, respectively. There is about 2 mm distance between the sensitive element and the ribbon during the test. The results are shown in Figure 9. It is clear from Figure 9a that the GMI curves move to a lower field due to the presence of the ribbon in front of GMI sensor, the larger the degree, the lower field the curve moves to. This can be understood as follows: the stray magnetic field arising in front of the GMI sensor strengthens the external magnetic field, the larger the degree, the stronger the stray magnetic field. It is easy to speculate that the GMI-field curve should move to a lower or higher magnetic field when the ribbon is rotated at different degrees. 

**Figure 10:**
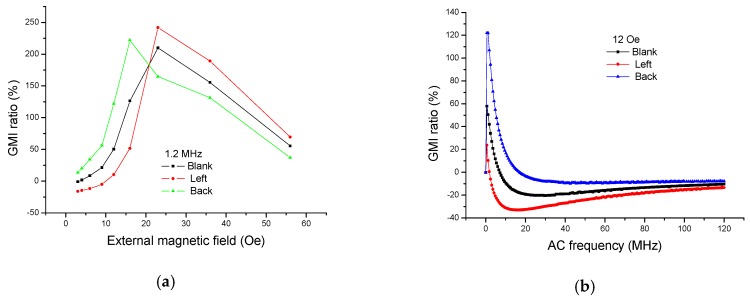
The giant magnetoimpedance responses when the ribbon (3 × 5 mm^2^) is detected at the back and on the left of the giant magnetoimpedance sensor II: (**a**) field dependence and (**b**) frequency dependence.

We have also measured the GMI response when the ribbons are, respectively, tested on the left and at the back of the GMI sensor II, respectively, as shown in Figure 10. It is clear from Figure 10a that the GMI curve moves to a lower magnetic field due to the presence of the ribbon at the back of GMI sensor. On the contrary, the GMI curve moves to a higher magnetic field due to the presence of the ribbon on the left side of the GMI sensor. The above phenomena can also be explained by the superimposed effect of the magnetic fields. Since the sensitive elements (meander-line sandwich structure) are symmetrical, it is easy to speculate that the GMI curve should move to a higher magnetic field when the ribbon is tested on the right or on the bottom of the GMI sensor. In fact, the permeability of the soft magnetic material is determined by the effective magnetic field (*H*_eff_) inside the material. Since the demagnetizing field *H_d_* inside the material can weaken the external magnetic field, the effective magnetic field (*H*_eff_) inside the material can be expressed as *H*_eff_ = *H*_e_ – *H*_d_. When the ribbons are tested on the top of the GMI sensor, the stray magnetic fields (*H*_s_) of ribbons can partly cancel out the external magnetic field as well. Thus, the effective magnetic field inside the material can also be expressed as *H*_eff_ = *H*_e_ – *H*_d_ – *H*_s_. As the GMI ratio reaches the peak when *H*_eff_ is equal to the anisotropy, and *H*_d_ remains constant, the presence of *H*_s_ shifts the peak to a higher external magnetic field. The larger area of the ribbon, the larger the stray magnetic field is, and the larger shift of the GMI peak. Similarly, it is easy to explain that the peak of GMI shifts to a lower external magnetic field. In summary, no matter which position the ribbon locates, one might anticipate that there should be one conclusion: the GMI curve (field dependency) should shift to a lower magnetic field or a higher magnetic field due to the reinforcing or undermining effect of the stray magnetic fields. At the moment, it is impossible for us to perform numerical simulations. However, it is expected that an accurate theoretical model will be built to simulate the disturbing effect of the stray magnetic fields in future work.

## 4. Conclusions

The disturbing effect of the stray magnetic fields in Fe-based amorphous ribbons has been examined by a meander-line GMI sensor. We suggest that the presence of the ribbons in front of GMI sensor has strengthened the longitudinal external magnetic field. Thus, the GMI curve shifts to a lower external magnetic field. On the contrary, the presence of the ribbons on the top of the GMI sensor has partly canceled out the longitudinal external magnetic field, and thus the GMI curve shifts to a higher external magnetic field. The larger the area, the stronger the stray magnetic field, the larger movement to which the GMI curve shifts. We also found that the GMI curve moves to a lower or higher magnetic field when the ribbon is rotated at different degrees. The obtained results can provide guidance for development of GMI sensors in detecting the general stray magnetic fields of the ferromagnetic equipments in different positions and angles.
